# The Treatment of Metastatic Non-Small Cell Lung Cancer in the Elderly: An Evidence-Based Approach

**DOI:** 10.3389/fonc.2014.00178

**Published:** 2014-07-10

**Authors:** David E. Dawe, Peter Michael Ellis

**Affiliations:** ^1^Section of Hematology and Medical Oncology, Department of Internal Medicine, University of Manitoba, Winnipeg, MB, Canada; ^2^Department of Oncology, McMaster University, Hamilton, ON, Canada

**Keywords:** non-small cell lung cancer, chemotherapy, elderly, geriatric assessment, targeted therapy

## Abstract

An increasing proportion of patients with advanced non-small cell lung cancer (NSCLC) are over 70 years old, raising unique challenges for treatment decision-making. While these patients are underrepresented in clinical trials, there is an emerging body of evidence associated with this group. The lesson of comprehensive geriatric assessment is that chronological age does not always correlate with physiological age and a variety of important co-morbidities and geriatric syndromes can go undetected in a typical history and physical. These co-morbidities and expected physiologic changes due to aging complicate decision-making around appropriate treatment. This review discusses geriatric assessment in elderly cancer patients and evaluates the current evidence for chemotherapy and targeted therapy for patients with advanced NSCLC aged ≥70 years.

## Introduction

The number of seniors in Canada is projected to more than double between 2005 and 2036 (1) and global life expectancy has increased continuously over the last 40 years (2). Forty-three percent of cancers in 2010 were diagnosed in patients 70 years or older (3). Therefore, barring a significant change in cancer incidence, the absolute number of cancers diagnosed in elderly patients can be expected to increase substantially both in Canada and worldwide.

Worldwide, lung cancer is the leading cause of cancer-related mortality and by 2010 was the fifth overall leading cause of death (4, 5). Eighty-five percent of diagnosed lung cancer patients have non-small cell lung cancer (NSCLC) (6, 7). The median age of diagnosis is 70 years and has been increasing (7, 8). Lung cancer is therefore a disease of older adults and up to 70% of patients are diagnosed in advanced stage, where the standard treatment is systemic therapy (8).

This advanced age is an important treatment consideration due to the complex interplay of physiologic changes associated with aging, co-morbidities, competing mortality, and potential differences in priorities among younger vs. older individuals when prognosis is limited. These issues are compounded by difficulty in predicting both benefit from chemotherapy and risk of toxicity in older patients due to historical underrepresentation in clinical trials (9, 10).

This narrative review will discuss the complexity of treating geriatric patients and outline the current state of evidence for the use of chemotherapy in this population for the treatment of advanced lung cancer.

## Physiologic Changes

Physiologic changes with aging occur in a number of organ systems that can affect the safety of chemotherapy (Table 1). Glomerular filtration rate is typically estimated to decrease by 1 mL/min/year beyond age 40 (11–14). In addition to this reduction in renal clearance, there is also impairment in the handling of water and electrolytes (13, 15). These changes can increase the risk to elderly lung cancer patients for toxicity from drugs primarily cleared by the kidneys, as well as dehydration and electrolyte imbalances.

**Table 1:** **Physiologic changes with aging**.

Organ system	Changes	Effect on chemotherapy
Renal	Decreased glomerular filtration	Decline in renal drug clearance that increases risk of drug toxicity
	Impaired water and electrolyte handling	Increased risk of dehydration
Gastrointestinal	Decreased gastric blood flow and delayed gastric emptying	Variable drug absorption
	Decreased absorptive capacity	Decreased absorption of oral drugs
	Decreased mucosal repair	Vulnerability to mucositis
Hepatobiliary	Decreased liver mass and blood flow	Reduced hepatic metabolism
	Reduction in cytochrome P450 activity	Greater vulnerability to P450 associated with drug interactions
Body composition	Increased fat and decreased water	Changes drug volume of distribution
Hematologic	Decreased marrow cellularity, proliferation, and mobilization	Impaired response to cytopenias, delayed blood count recovery, and higher risk of infection

The gastrointestinal system also changes with age, affecting drug absorption and the risk of mucositis (16, 17). Inconsistency in absorption results from reduced gastric blood flow, delayed gastric emptying, and a reduction in intestinal absorptive capacity (18–21). The vulnerability to mucosal injury arises from alteration of protective mechanisms, including a reduction in mucus and bicarbonate secretion (18). More importantly, elderly individuals generally show a decrease in hepatic mass and blood flow, which reduces drug metabolism (22). A reduction in activity of the cytochrome P450 system can also occur, resulting in a higher risk of drug interactions (23). The changes in metabolism can be further exacerbated by body composition changes that increase fat content and decrease water composition, thereby altering the volume of distribution for many drugs (24).

Finally, important changes occur in the bone marrow, with decreased cellularity, precursor proliferation, and cell mobilization (25, 26). These changes result in decreased bone marrow reserve. This altered bone marrow responsiveness increases the risk of marrow suppression and associated complications from chemotherapy and can delay further treatment administration (27, 28).

## Prediction of Toxicity

These physiologic shifts can increase the risk of chemotherapy toxicity in older individuals. However, clinical experience identifies many patients who seem much younger (or older) than their chronological age. This heterogeneity was strikingly demonstrated through comparison of life expectancies within geriatric age groups. Life expectancy for a 75-year-old woman ranged from 6.8 years (lowest 25th percentile) to 17 years (highest 25th percentile) and the same values for a man are 4.9 and 14.2 years (29). This variation in life expectancy reflects differences in baseline health, comorbidity, and genetics (30). It seems reasonable to hypothesize that the individual with better life expectancy has less risk of toxicity and more chance of benefit from chemotherapy, since they have less risk of competing causes of mortality. The challenge is identifying these patients and improving the up to 44% of lung cancer patients ≥70 years, who may require hospitalization during chemotherapy (31).

### Comprehensive geriatric assessment

Historically, physicians used a combination of performance status (PS), measured using the Eastern Cooperative Oncology Group (ECOG) PS scale, and organ function as determined through blood work to determine, which patients qualified for chemotherapy treatment (32). This approach has been demonstrated to perform poorly when compared to more formal geriatric assessment (33–35). Comprehensive geriatric assessment (CGA) is usually composed of medical, functional, mental, social, and nutritional assessments, as well as explicit assessment of prescription drug use (36). A variety of studies have been completed to evaluate the usefulness of CGA in oncology patients. Systematic reviews of the available evidence show that CGA identifies problems that would otherwise be missed, leads to modifications in treatment plans, and helps predict toxicity from chemotherapy (37–42). Modification of treatment plans occurs in 21–53% of patients, suggesting that oncologists believe the additional information is valuable (42). CGA is also better than physician opinion for identifying frail elderly patients who experience greater toxicity (43). When tested in elderly NSCLC patients, CGA was feasible (44–46). Frail patients also exhibited poorer survival (46). However, when Corre et al. allocated patients to treatment based on CGA, survival was not different between groups, but toxicity was reduced in the arm allocating treatment using CGA (47).

### Other predictive tools

While a CGA can be very useful, it has not become a routine part of oncologic care because it is time and labor intensive. The mean duration of CGA during one prospective study was 80 min/patient (48). Such a time commitment is difficult to undertake in lung cancer patients with metastatic disease, since it may delay patient throughput in clinic and/or delay commencement of treatment, a serious concern when patients have an average life expectancy of 10–12 months (8). In light of these concerns, a number of groups have attempted to shorten the CGA or provide a screening tool. Two groups have published new tools geared toward predicting chemotherapy toxicity and derived from multivariable analyses of CGAs conducted in cancer patients (Table 2) (33, 49). The chemotherapy risk assessment scale for high-age patients (CRASH) is actually composed of two scores, one for hematologic toxicity and another for non-hematologic toxicity (49). Diastolic blood pressure, instrumental activities of daily living (IADL), lactate dehydrogenase, and a proprietary Chemotox score help stratify the likelihood of experiencing Grade 3–4 hematologic toxicity into low (7%), medium-low (23%), medium-high (54%), and high (100%). For non-hematologic adverse events, the predictors are: ECOG PS, mini mental status, mini nutritional assessment, and the Chemotox score. The same predictive categories for non-hematologic toxicity predict risks of 33, 46, 67, and 93% (49). The Chemotox score is a quantitation of the toxicity of chemotherapy regimens that the group developed previously (50, 51). The cohort used for the CRASH score contained 21% lung cancer patients. The Cancer Aging Research Group (CARG) derived another predictive tool (33, 52). The CARG score uses 11 factors to stratify risk: age ≥72, cancer type, standard chemotherapy dosing, polychemotherapy, low hemoglobin, low creatinine clearance, fair or worse hearing, falls, needing help with medications, trouble walking 1 block, and decreased social activity. This score classified lung cancer patients into low (10%), intermediate (40%), or high (60%) risk of Grade 3–5 toxicity (52). This score was better able to stratify risk of toxicity than Karnofsky PS alone. Both of these predictive tools are exciting because they provide information that can be used to discuss chemotherapy treatment with elderly patients. Unfortunately, neither has been validated outside of the initial population and, therefore, widespread adoption is not yet justified.

**Table 2:** **Significant factors in scores predicting chemotherapy toxicity**.

CRASH score (49)	Hematologic	Non-hematologic
	Diastolic blood pressure	ECOG performance status
	IADL	Mini mental status
	LDH	Mini nutritional assessment
	Chemotox score	Chemotox score

**CARG score (33)**	**Predictive factors**

	Age ≥72 years
	Standard chemotherapy dosing
	Multi-drug chemotherapy
	Low hemoglobin
	Low creatinine clearance
	Decreased hearing
	Fall within 6 months
	Needs help taking medications
	Limited in walking 1 block
	Decreased social activity	

*IADL, instrumental activities of daily living; LDH, lactate dehydrogenase; ECOG, Eastern Cooperative Oncology Group*.

## Evidence for Chemotherapy

Since 1995, standard first line chemotherapy for younger patients with stage IV NSCLC has been a platinum doublet. The meta-analysis supporting this recommendation demonstrated a 10% improvement in 1-year survival for patients treated with chemotherapy compared to supportive care (53). However, the first randomized trial focusing specifically on elderly patients was not published until 1999 (54, 55). Since then, only a few randomized trials have been conducted in patients 70 years and older with metastatic NSCLC. Most recommendations have been based on subgroup analyses or cohort studies. This lower level of evidence has likely contributed to uncertainty among health professionals regarding the standard of care in these patients.

The primary trial investigating the utility of single-agent chemotherapy compared to the best supportive care in elderly patients with metastatic NSCLC was the Elderly Lung Cancer Vinorelbine Italian Study (ELVIS) (55). The experimental arm of this RCT was single-agent vinorelbine (30 mg/m^2^) administered on days 1 and 8 of a 21-day cycle. This treatment resulted in an improvement in median survival (28 vs. 21 weeks) and 1-year survival (32 vs. 14%), in addition to improvement in some lung cancer symptoms. Despite falling short of its 350 patient accrual target and closing prematurely, this established a new standard of care, which was incorporated as the control arm in further studies.

### Doublet chemotherapy

Subsequent trials have evaluated doublet chemotherapy regimens. Due to concerns about toxicity, these trials initially examined non-platinum chemotherapy combinations. The Southern Italy Cooperative Oncology Group (SICOG) conducted an RCT comparing gemcitabine and vinorelbine in combination to vinorelbine alone (56). The combination arm reported a median survival of 29 weeks compared to 18 weeks for vinorelbine alone. While this difference was statistically significant, there were concerns that the control group had worse survival than expected. These concerns prompted the multicenter Italian lung cancer in the elderly study (MILES), which compared three arms: vinorelbine plus gemcitabine, gemcitabine alone, and vinorelbine alone (57). Whereas, the SICOG trial enrolled 120 patients, MILES randomized 698 patients between the three arms. Median survivals were 30, 28, and 36 weeks for each of the arms, respectively. There was no statistically significant difference. There was, however, greater toxicity in the combination arm, specifically for neutropenia, thrombocytopenia, anemia, vomiting, constipation, and hepatic toxicity (57). These results do not support the use of the combination of vinorelbine and gemcitabine.

Further evaluation of doublet chemotherapy in patients ≥70 years was pursued. In one RCT of elderly patients or those with ECOG PS 2, the combination of gemcitabine and paclitaxel improved median survival to 9.2 months compared with 5.1 months for gemcitabine alone (58). However, a RCT in the same mixed population comparing gemcitabine/docetaxel to weekly docetaxel reported no difference in survival (59). A systematic review with meta-analysis of RCTs comparing non-platinum doublets with single-agent therapy for elderly patients showed no survival advantage to doublet therapy and higher risk of thrombocytopenia (60). Perhaps the most promising regimen was the combination of carboplatin with paclitaxel. One phase II trial demonstrated that weekly paclitaxel combined with carboplatin resulted in a 14-month median survival with quite manageable toxicity (61). Interestingly, when compared to standard paclitaxel, weekly paclitaxel appears to have equivalent benefit, but reduces the risk of neutropenia and peripheral neuropathy (62). The landmark trial investigating the use of platinum doublets in the elderly is Intergroupe Francophone Cancérologie Thoracique (IFCT)-0501 (63). This trial included 451 patients aged 70–89 years, with locally advanced or metastatic NSCLC and a PS of ECOG 0–2. Patients were randomized to carboplatin and weekly paclitaxel vs. monotherapy with either gemcitabine or vinorelbine. The trial was stopped early after interim analysis demonstrated superiority for the doublet regimen. Median overall survival was 10.3 months for carboplatin and paclitaxel compared to 6.2 months for monotherapy (hazard ratio 0.64, 95% CI 0.52–0.78, *p* < 0.0001). The largest increases in toxicity for doublet chemotherapy were neutropenia (48.4 vs. 12.4%) and asthenia (10.3 vs. 5.8%) (63). Point estimates were quite consistent for all subgroups and multivariable analysis confirmed expected prognostic factors like sex, PS, adenocarcinoma histology, and smoking history.

One final trial, conducted in Japan by Takeda et al., has only been published in abstract form (64). The trial enrolled 276 patients, who were chemotherapy naïve, age >70 years, ECOG PS 0–1, and with stage III/IV NSCLC. Patients were randomized to receive either docetaxel every 3 weeks or weekly cisplatin–docetaxel. Enrollment was stopped early due to futility, with median survival times of 13.3 months for cisplatin–docetaxel and 17.3 months for docetaxel alone (hazard ratio 1.557, 95% CI 0.976–2.485). Interestingly, neutropenia was far more common with docetaxel alone than the doublet regimen 88 vs. 11%. The survival of the monotherapy group was remarkably high compared previous trials (65–67).

The majority of subgroup analyses from earlier trials suggest that survival is similar, though not always equal, between younger and older patients with advanced NSCLC who receive the same chemotherapy (68–71). The evidence suggests that the results of IFCT-0501 should form the standard of care for first line chemotherapy treatment in fit elderly patients with advanced NSCLC, especially when no molecular abnormalities are detected.

In the second line setting, there are no elder-specific trials. A retrospective analysis of the JMEI trial comparing docetaxel to pemetrexed was completed for patients ≥70 years old vs. younger patients. Median survival of 9.5 and 7.7 months was reported for elderly patients receiving pemetrexed (*n* = 47) and docetaxel (*n* = 39). In younger patients, these values were 7.8 and 8.0 months. Febrile neutropenia occurred in only 2.5% of elderly patients receiving pemetrexed, but 19% of those being treated with docetaxel (*p* = 0.025) (72).

## Targeted Therapies

### Bevacizumab

Bevacizumab is a monoclonal antibody that targets vascular endothelial growth factor (VEGF) and can be used in combination with first line platinum-based chemotherapy. Two trials, ECOG 4599 and AVAiL, originally tested the addition of bevacizumab to standard chemotherapy (73, 74). Analyses of the elderly patients in both of these trials were conducted. The AVAiL trial, comparing cisplatin and gemcitabine with or without bevacizumab, showed no improvement in overall survival in the elderly population (*n* = 304), with the addition of bevacizumab (73). An analysis of patients ≥70 years in the ECOG 4599 trial of carboplatin and paclitaxel with or without bevacizumab reported overall survival was 11.3 months with bevacizumab and 12.1 months without. There was a higher incidence of bleeding, neutropenia, and proteinuria in older compared to younger patients (74). There does not appear to be compelling evidence to include bevacizumab for those older than 65–70 years of age.

### *EGFR* tyrosine kinase inhibitors

Molecularly defined subtypes of NSCLC have become incredibly important to management over the last 5 years. Epidermal growth factor receptor (EGFR) mutations are detected in approximately 15% of Caucasian patients with advanced NSCLC and these mutations are found more often, but not exclusively in younger, never smoking women, or those of Asian ethnicity (75). The presence of an *EGFR* mutation is highly predictive of benefit from EGFR tyrosine kinase inhibitors (TKIs) (76). More widespread screening of all NSCLC tumor samples for molecular abnormalities will increase the number of *EGFR* mutations identified in the elderly. Available data demonstrate EGFR TKIs (erlotinib, gefitinib, or afatinib) result in better progression free survival (PFS) and favorable toxicity compared to chemotherapy in NSCLC patients with an *EGFR* mutation (76–79). While few studies have examined the effect of this strategy exclusively in elderly patients, available data would suggest that elderly patients have similar response rate and PFS (80–84). Toxicities reported were the expected diarrhea, rash, and risk of transaminitis.

The NCIC BR.21 trial evaluated erlotinib in NSCLC patients who progressed after one or two prior chemotherapy treatments regardless of *EGFR* mutation status. The improvement in overall survival was seen in both *EGFR* mutated and wild type patients. A retrospective analysis of treatment effect and age in BR.21 found no statistically significant difference in treatment effect between younger and older patients for overall survival. Elderly patients did experience more Grade 3–4 toxicity (35 vs. 18%, *p* < 0.001). Based on this subgroup analysis, erlotinib seems to be a reasonable option for elderly patients in the second or third line setting (85). A trial in vulnerable elderly patients by CGA adds further support to this opinion, since both gemcitabine followed by erlotinib or the reverse on progression showed similar survival and tolerability (45).

### ALK tyrosine kinase inhibitors

The other actionable mutation found in NSCLC is a translocation in echinoderm microtubule associated protein-like 4 – anaplastic lymphoma kinase (*EML4–ALK*) gene, which is found in approximately 4% of patients with adenocarcinoma (75). Data in younger patients have been extremely promising with the use of crizotinib for *EML4–ALK* translocated NSCLC (86–88). Few patients were older than 70 years. A phase I study including 149 patients did report a response rate of 65% (40.8–84.6%) in patients ≥65 years (87). More recently, two other early-phase clinical trials with different ALK TKIs demonstrated response rates >50%, with ceritinib showing impressive responses even in crizotinib resistant disease (89, 90). While there are little data in elderly patients, there is no reason to believe this group would derive less benefit from ALK TKI therapy.

## Conclusion

An increasing proportion of patients with advanced NSCLC are over 70 years old, raising unique challenges for treatment decision-making. While these patients are underrepresented in clinical trials, there is an emerging body of evidence associated with this group. The lesson of CGA is that chronological age does not always correlate with physiological age and a variety of important co-morbidities and geriatric syndromes can go undetected in a typical history and physical. Geriatric assessment provides medical oncologists with information that can affect treatment decision and help predict chemotherapy toxicity. Abbreviated CGAs or newly derived tools offer the promise of more widespread implementation of appropriate assessment of elderly patients.

For patients fit enough to consider first line chemotherapy, a platinum doublet appears to be a reasonable standard of care. Adding bevacizumab does not appear to improve overall survival. In the second line, pemetrexed, docetaxel, or erlotinib are all options for consideration. Pemetrexed would be the preferred option for patients with non-squamous histology. For patients with *EGFR* mutated disease, using an EGFR TKI as a first line treatment is a reasonable approach, though there is little evidence specific to elderly populations.

Further research is needed on the validation of tools that predict chemotherapy toxicity and prognosis to facilitate informed consent and treatment decisions. More studies focusing on elderly patients are also essential to help account for the physiologic changes inherent in this population. As we move forward, medical oncology is becoming geriatric oncology in many ways.

## Conflict of Interest Statement

Dr. David E. Dawe has no conflicts of interest to report. Dr. Peter Michael Ellis reports having received honoraria from Lilly, Roche, and Boehringer Ingelheim for advisory boards.
